# Machine learning in physical activity, sedentary, and sleep behavior research

**DOI:** 10.1186/s44167-024-00045-9

**Published:** 2024-01-30

**Authors:** Vahid Farrahi, Mehrdad Rostami

**Affiliations:** 1 Institute for Sport and Sport Science, TU Dortmund University, Dortmund, Germany; 2 Centre of Machine Vision and Signal Analysis, Faculty of Information Technology, University of Oulu, Oulu, Finland

**Keywords:** Wearables, Supervised learning, Unsupervised learning, Classification, Clustering, Machine learning modelling, Predictive modelling

## Abstract

The nature of human movement and non-movement behaviors is complex and multifaceted, making their study complicated and challenging. Thanks to the availability of wearable activity monitors, we can now monitor the full spectrum of physical activity, sedentary, and sleep behaviors better than ever before—whether the subjects are elite athletes, children, adults, or individuals with pre-existing medical conditions. The increasing volume of generated data, combined with the inherent complexities of human movement and non-movement behaviors, necessitates the development of new data analysis methods for the research of physical activity, sedentary, and sleep behaviors. The characteristics of machine learning (ML) methods, including their ability to deal with complicated data, make them suitable for such analysis and thus can be an alternative tool to deal with data of this nature. ML can potentially be an excellent tool for solving many traditional problems related to the research of physical activity, sedentary, and sleep behaviors such as activity recognition, posture detection, profile analysis, and correlates research. However, despite this potential, ML has not yet been widely utilized for analyzing and studying these behaviors. In this review, we aim to introduce experts in physical activity, sedentary behavior, and sleep research—individuals who may possess limited familiarity with ML—to the potential applications of these techniques for analyzing their data. We begin by explaining the underlying principles of the ML modeling pipeline, highlighting the challenges and issues that need to be considered when applying ML. We then present the types of ML: supervised and unsupervised learning, and introduce a few ML algorithms frequently used in supervised and unsupervised learning. Finally, we highlight three research areas where ML methodologies have already been used in physical activity, sedentary behavior, and sleep behavior research, emphasizing their successes and challenges. This paper serves as a resource for ML in physical activity, sedentary, and sleep behavior research, offering guidance and resources to facilitate its utilization.

## Introduction

Our daily lives constitute three main components—physical activity, sedentary, and sleep behaviors—that are interconnected with each other [1] and codependently related to various aspects of our health [2, 3]. In recent years, there has been a growing use of wearable devices to measure these behaviors, adding to the complexity of studying and understanding them. Nowadays, wearable activity monitors have the capacity to continuously capture the entire spectrum of movement and non-movement behaviors over extended periods [2], lasting even up to several weeks [4]. However, making sense of such data is challenging and often requires the use of advanced analytical tools tailored to handle their complex nature [2, 3].

Physical activity, sedentary, and sleep behavior data often fail to conform to the assumptions of classical statistical methods. The complex interactions and co-dependencies among these movement and non-movement behaviors across the 24-h day make it unclear what type of functional relationship one should use to describe such data mathematically [3, 5]. Additionally, numerous other complex issues, such as correlates and determinants [6], tailored recommendations [1], and activity recognition from activity monitors [7, 8], require more innovative analytical approaches. Nevertheless, human behaviorists and epidemiologists who are interested in monitoring these behaviors and assessing their impact on health and diseases have traditionally relied on classical statistical methods, such as linear regression analysis, to manage and analyze these complex datasets [2, 3, 9].

Owing to their underlying mathematical principles, machine learning (ML) methods can handle complex and high-dimensional datasets [10, 11]. In essence, ML and statistics share many similarities, but they approach data in different ways [12, 13]. Statistics is typically used to understand and interpret data. It often deals with smaller, more controlled datasets and focuses on understanding relationships between variables, testing hypotheses, and making inferences about a population based on a sample. ML, on the other hand, is more like a data-driven problem-solving approach [12, 13]. It involves teaching machines to learn from data without explicitly programming them. This gives ML algorithms the capability to improve their performance over time as they are exposed to more data. In a more formal definition, ML fundamentally represents a subfield of artificial intelligence and incorporates a wide array of methodologies aimed at identifying and learning patterns from data. Unlike traditional statistical approaches that focus on hypothesis testing and parameter estimation, ML methods are more concerned with predictive modeling and pattern recognition [11, 14].

However, despite the recent trend towards employing more complex statistical methods [15–17], ML has not yet gained widespread adoption for analyzing such datasets [3]. This may be partly because researchers interested in analyzing and studying human movement and non-movement behaviors often lack prior experience in ML, which can make approaching this subject area overwhelming and subsequently hinder its applications. Such approaches offer a valuable set of data modelling techniques that complement those found in classical statistics [10, 18], making them well-suited for analyzing and studying physical activity, sedentary, and sleep behavior data.

In the physical activity, sedentary, and sleep behavior context, ML can help be useful in a number of ways, particularly after the recent shift towards assessment of these behaviors with wearable devices. Whether it involves identifying patterns of physical activity in large populations [16, 19], classifying individuals based on their sedentary behavior patterns [20], or understanding the interrelationships between sleep duration, variability, or quality [21], ML stands as a powerful tool for exploring and deriving insights from diverse and voluminous datasets. Yet, while the application of ML does not require extensive expertise in computer science or mathematics, employing it without a proper understanding can lead to biased models and findings that may misrepresent reality. Some argue that even practicing ML can be as straightforward as performing linear regression analysis [22]—a method commonly used by many researchers working with data related to physical activity, sedentary, and sleep behaviors. This realization can be enlightening for those who perceive ML solely as complex analytical methods.

The aim of this study is to provide an overview of ML modeling techniques, which are typically discussed in technical journals and books. To assist researchers working with data on physical activity, sedentary, and sleep behaviors, we offer a step-by-step guide outlining the typical steps taken when performing ML modeling. Our guide begins by outlining the general steps involved in ML modeling. We then introduce both supervised and unsupervised learning, along with several commonly used algorithms for these two types of ML. This is followed by a brief overview of the literature covering three research domains where ML has been successfully applied in physical activity, sedentary, sleep behavior research. We conclude with a discussion on the more practical aspects of ML, including tools for executing ML modeling and the limitations of ML techniques.

## Machine learning modeling

**Fig. 1:**
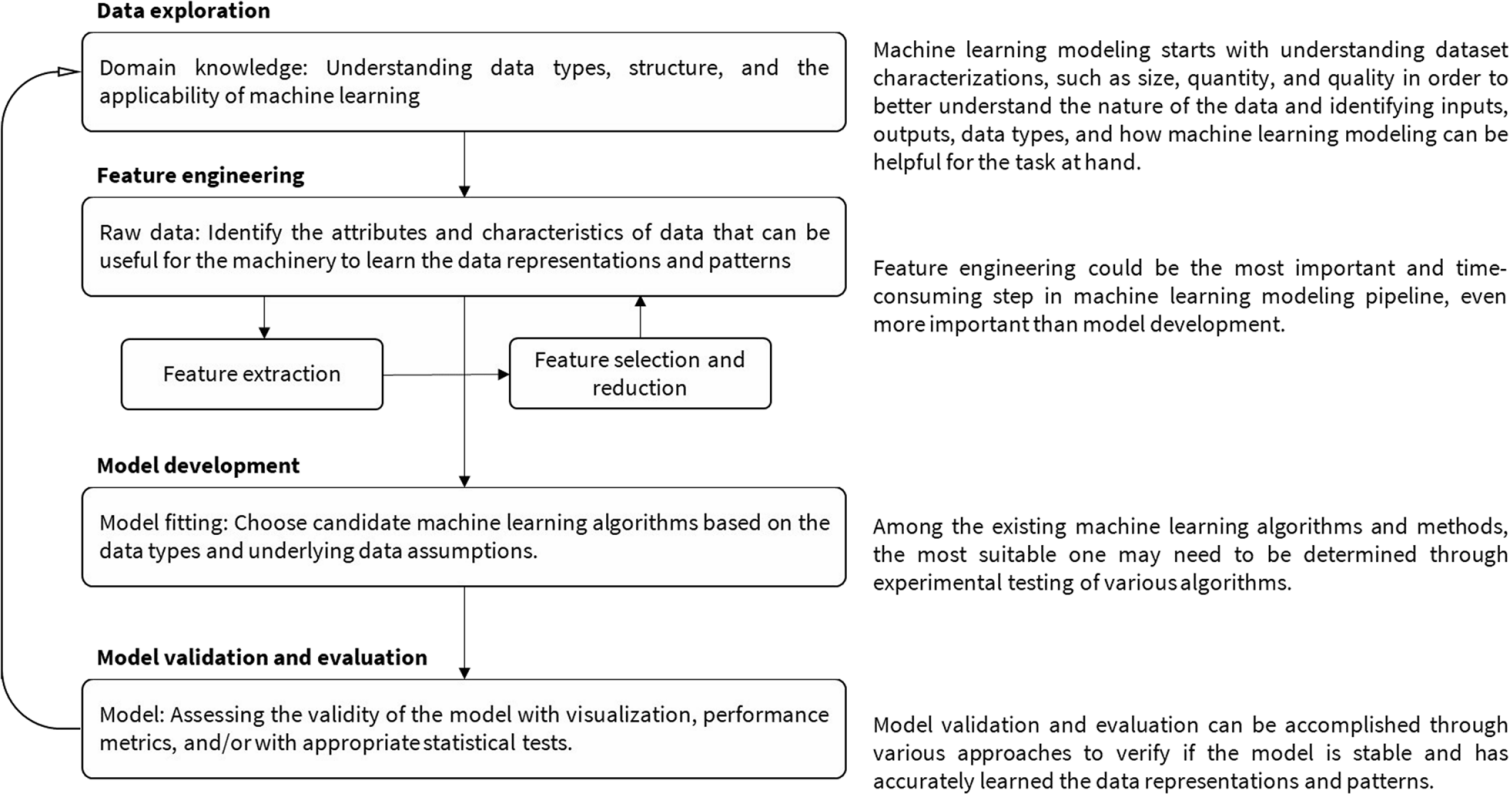
The recursive and sequential process of machine learning modelling pipeline

ML can handle a vast array of tasks, such as classifying observations into predefined categories, clustering data into groups with shared characteristics, and regressing outcomes against multiple factors to understand their individual contributions [10, 11]. These tasks are typically undertaken using one of two primary ML approaches: supervised learning or unsupervised learning [10, 23]. The ML modeling process for both types is iterative, consisting of a series of sequential steps, often organized into what is known as an ML pipeline [10, 24]. To follow this structure, we first examine the four essential stages of the ML modeling process: (1) data exploration, (2) feature engineering, (3) model development, and (4) model validation and evaluation. After outlining these steps, we introduce supervised and unsupervised learning. Figure 1 provides a bird’s-eye view of the general steps involved in ML modeling.

### Data exploration

ML modelling typically begins with the exploration of data [24]. In this phase, it is common to investigate various aspects of the dataset, such as its size, structure, quantity, and quality. Often, data visualizations are utilized to discern trends, identify outliers, and develop an understanding of the data’s inherent characteristics. These initial steps are crucial for acquiring a better domain knowledge, which, in turn, enables ML practitioners to make informed decisions at every stage of designing and applying ML models to the problems at hand. Domain knowledge is important for feature engineering and can even be incorporated in ML algorithms to improve the transparency of ML, aligning more closely with how experts in the field operate [25].

### Feature engineering

Features are the attributes or characteristics of the data that are needed for performing any ML task (supervised or unsupervised) [26, 27]. Feature engineering is the link between raw data with no apparent meaningful pattern and successful ML model development, and therefore can have a substantial impact on the ML modelling [26, 28]. Typically, this process involves a considerable amount of data cleaning, preprocessing, transformation, and visualization, making it the most labor-intensive step in ML modeling—often surpassing the time required to fit algorithms to the data [29]. Feature engineering generally encompasses two key phases: feature extraction and feature selection or reduction, as illustrated in Fig. 1.

Feature extraction refers to the process of converting raw data into meaningful features, or simply measuring different characteristics and aspects of raw data, whereas feature selection and reduction is the process of choosing, transforming, and shaping discriminative features for ML modelling [26, 28, 29]. More precisely, feature selection and reduction involve the careful curation of a subset of features from an initially expansive list of possibilities created during feature extraction. During feature engineering, the primary goal is to retain only the most pertinent and informative attributes while disregarding redundant or less influential ones [26, 28, 29].

In the context of physical activity, sedentary, and sleep behaviors, feature engineering remains a fundamental and crucial step in characterizing and analyzing such data using ML methodologies; whether ML is utilized for activity recognition from wearable data [28] or for studying behavior patterns within individuals or populations [20, 30]. The features represent the quantifiable attributes that hold essential information, for example about an individual’s movement patterns, energy expenditure, and lifestyle habits. To provide an example, when dealing with physical activity data, these features could be a wide array of parameters such as step counts, heart rate variability, duration of physical activity, intensity levels, and even contextual information like location and time of day. On the other hand, when focusing on sedentary behaviors, important features might include metrics related to sitting time, screen time, posture, and breaks from sedentary activities.

### Model development

The iterative process of developing and validating ML models necessitates careful consideration in the choice of algorithms, which depends on the types of data available and their underlying assumptions. Typically, the best-performing ML algorithm is identified experimentally in an iterative manner [28, 31, 32]. Take, for instance, the prediction of activity types and intensities from wearable devices, which could logically involve analyzing sequential data due to the inherent sequential nature of human movement and non-movement behaviors. Given this assumption, when tackling an activity prediction problem with ML, prioritizing algorithms capable of processing sequential information may be more reasonable than potentially other alternatives [33].

### Model validation and evaluation

Once the model is fitted, the next step is to validate and evaluate the model. This essential phase involves a series of rigorous tests and assessments to ensure the model’s reliability and performance meet the desired standards. It is important to note that the impact of prior steps in the ML modeling pipeline, including feature engineering, is assessed and evaluated during the model validation and evaluation [10, 24].

## Supervised methods

Like traditional statistical methods (e.g., generalized linear models), supervised learning methods aim to identify the relationship between an outcome and a set of explanatory variables. The primary objective of supervised learning is to acquire a mapping from input data to corresponding outputs so that the model can make accurate predictions on new, unseen data [23]. Supervised learning can be done both for regression (continuous) and classification (categorical) tasks. However, unlike predefined model structures in traditional statistics, supervised learning leverages the data as a starting point [18, 23]. The ML machinery learns the mapping (predictive model) between a set of features and either a continuous target variable (regression) or a categorical target variable (classification) based on the patterns found in the data.

**Fig. 2:**
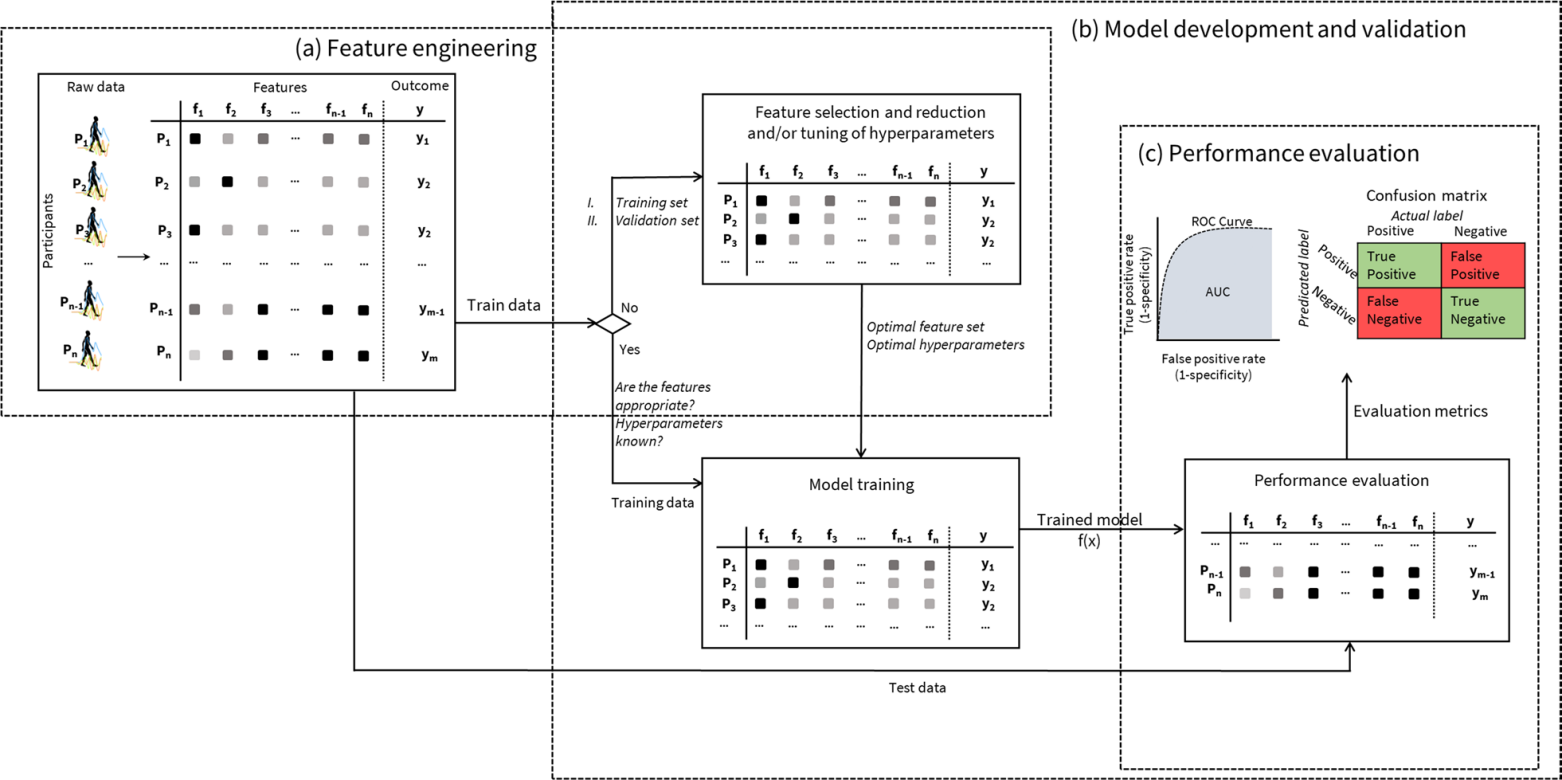
The process of supervised machine learning modelling. Following the feature engineering stage (**a**), once an appropriate feature set is determined, the next phase involves dividing the data into training and testing datasets (**b**). The training dataset is employed to construct the predictive model, while the testing dataset, which is not involved in the model building process, is reserved for assessing the expected predictive performance. In statistical terms, this process is analogous to drawing inferences about a population based on a finite and random sample. Given that the correct values for the target variables are known, it is possible to objectively verify the model’s performance (**c**)

**Fig. 3:**
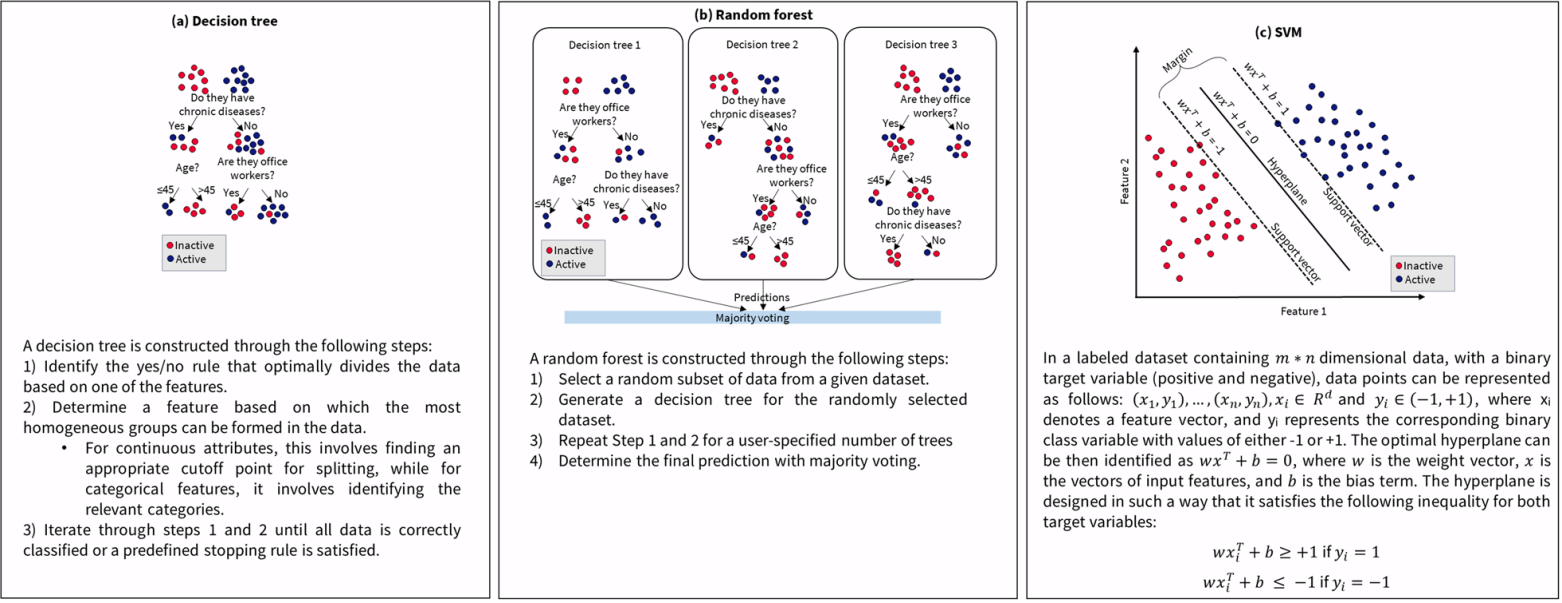
Three commonly employed machine learning algorithms with hypothetical data for predicting active and inactive individuals (**a**) A decision tree model comprising two levels. In decision trees, the selection of the appropriate attribute for each node at every level, the correct cut-off point, and the identification of relevant categories is typically accomplished using metrics such as Information Gain and Gini Index. **b** Random forest model comprised of three decision trees. **c** SVM model for a binary classification problem with two features

Figure 2 illustrates the general steps involved in the process of supervised learning for a classification task. For supervised learning, the machine needs to know the target variable for each observation. As illustrated in Fig. 2, the process of ML involves multiple interconnected steps, all working together to identify the most optimal model. There are a variety of supervised algorithms, each founded on different mathematical and statistical principles [23]. Below, we have briefly introduced decision trees, random forests, and support vector machines (SVM) that are widely used algorithms for supervised learning. These algorithms are illustrated in detail in Fig. 3.

### Decision trees

Decision trees are one of the popular ML methods employed for both classification and regression tasks [34]. Decision trees use a tree structure to represent the data, with each leaf node corresponding to a target variable and internal nodes representing attributes. They operate by recursively dividing the data into subsets based on the values of input features. The simplicity and interpretability of decision trees make them attractive, especially in health research [35, 36].

In a decision tree, the path from the root to a leaf node provides a clear and coherent rationale for each prediction. For example, physically active and inactive individuals can be classified by asking a series of simple yes/no questions (Fig. 3a). Decision trees are computationally inexpensive to train, evaluate and store. They can handle both categorical and continuous data and are fairly robust to outliers [35, 36]. However, they are often prone to overfitting (small changes in the training data set may lead to significantly different trees) and their simplicity may be a trade-off for relatively poor predictive performance compared to other ML algorithms [35, 36].

### Random forests

Random forests are an ensemble learning method that builds upon the decision tree algorithm [37]. Each decision tree is built through bagging, utilizing random subsets of the data and considering a random subset of features at each split. This ensemble technique may outperform the predictive ability of single decision trees, making it well-suited for a variety of supervised learning tasks [37, 38]. Figure 3b depicts the schematic of a random forest model built for classification of active and inactive individuals.

By combining the predictions of multiple trees, random forests reduce the risk of overfitting and improve the overall model’s generalization ability [37, 38]. However, if the dataset contains a lot of irrelevant features or noisy data, the random forest may include these data in the trees, potentially reducing its predictive performance. Random forests tend to be biased toward the majority class in imbalanced datasets [37].

### Support vector machine (SVM)

SVM is a supervised learning algorithm primarily employed for classification tasks [39]. The fundamental principle of SVM is to construct decision boundaries that effectively separate samples into predefined class categories. This decision boundary, also referred to as the hyperplane, is oriented in a manner that maximizes the distance from the closest data points of each class. SVM identifies these closest points, known as support vectors, which define the decision boundary and give the large marginal separation between the classes [39, 40]. Figure 3c illustrates the schematic of a SVM model created for binary classification problem.

SVM are particularly effective with high-dimensional data, which is often the case in analyzing physical activity, sedentary, and sleep behaviors. One of the key strengths of SVM is its use of kernel functions, which enable the algorithm to handle non-linear data by mapping it into a higher-dimensional space where a linear separation is possible [39, 40]. However, SVMs are known for their intensive memory usage, which can be a drawback when working with very large datasets. While SVM inherently addresses binary classification, multi-class problems are common in behavior analysis. To extend SVM to these problems, methods such as one-vs-all or one-vs-one are implemented, allowing the binary classifier to make decisions across multiple classes [39, 40].

### Supervised feature selection

**Fig. 4:**
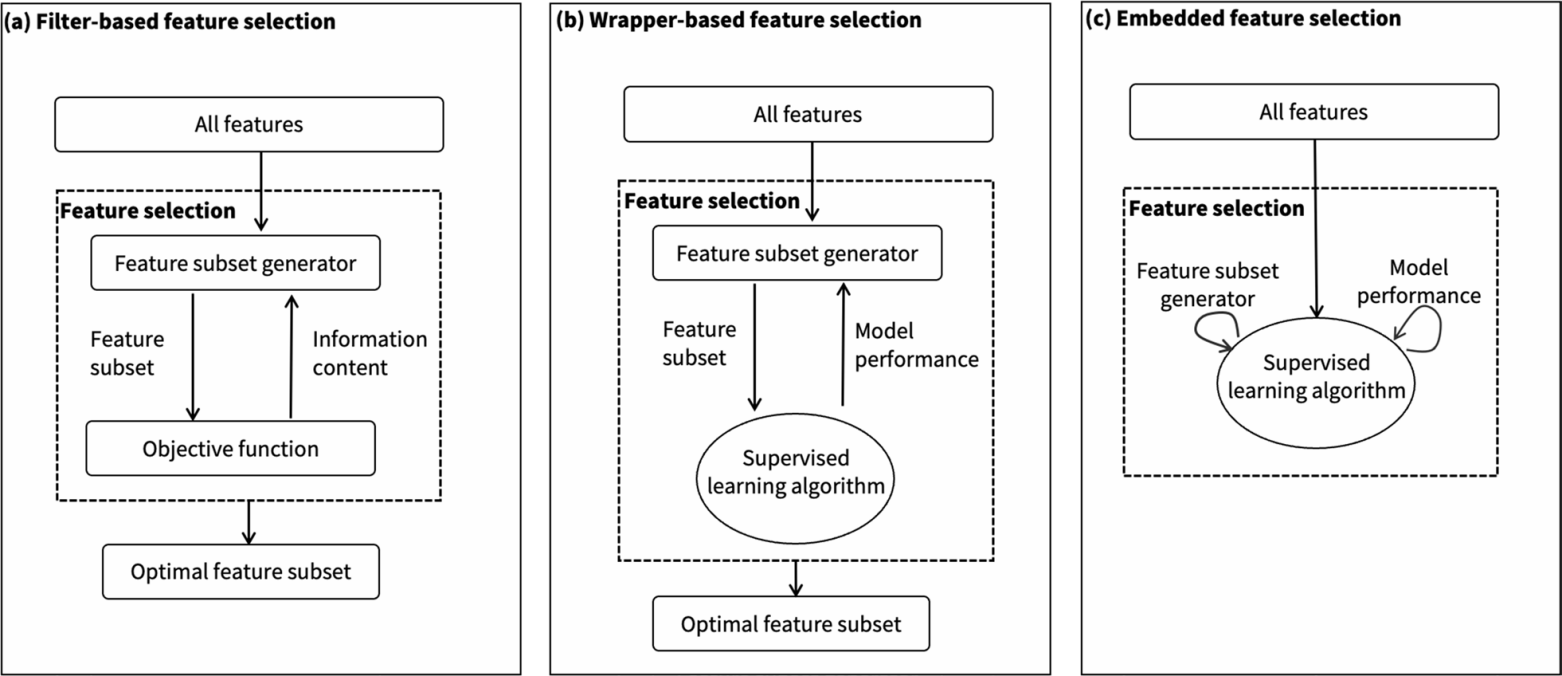
Three types of feature selection methods. **a** Filter-based feature selection employs an objective function to evaluate the relevance of a feature subset during the generation of feature lists. **b** Wrapper-based feature selection assesses the impact of a feature subset using the learning algorithm directly. **c** Embedded feature selection integrates an internal function to identify the most suitable feature subset for a prediction task

When the target variable is known, supervised feature selection methods can be employed. As demonstrated in Fig. 4a–c, these methods fall into three categories: filter, wrapper, and embedded methods [41, 42]. Filter methods assess the relevance and importance of all features based on predefined information-theoretic criteria. Relevance scores can be determined using various metrics such as distance metrics (e.g., Euclidean), correlation, mutual information, or consistency metrics, among others that have been proposed [43].

Wrapper methods employ a learning algorithm to evaluate the effectiveness of different subsets of candidate features [44]. Each subset is used to train a model, whose performance is then measured using a validation set or through cross-validation. Although wrapper methods are generally thought to yield better feature subsets compared to filter methods, they tend to be more prone to overfitting due to their dependence on the learning algorithm [44].

The embedded approach integrates the feature selection problem within the learning algorithm itself, with variable selection occurring during the model training process [41, 42]. This approach creates a direct interaction between feature selection and the learning process, unlike filter and wrapper methods. Decision trees and random forests are common examples of algorithms that have embedded feature selection mechanisms.

### Validation techniques

Validation techniques in supervised learning are methods used to evaluate the performance of a model on a separate dataset not used in training, to ensure it generalizes well to new, unseen data. Here, we discuss three validation techniques for supervised learning.

#### Data split

The data split, also known as the holdout method, divides the data into two subsets: a training set and a test set [45]. Initially, the model is trained on the training data and then applied to the test set to assess its predictive ability. In this approach, the test data serves as ‘unseen’ data, even though it originates from the same source. Data split validation may often include an additional subset called the validation set. The validation set is typically used to fine-tune hyperparameters in ML algorithm, before applying the model on the test set.

Data split technique should be used carefully when employed for ML modelling [45]. When dealing with a small dataset, the practice of splitting the data can be problematic as this will reduce the number of instances available for the algorithm to learn from. Splitting the data is also more vulnerable to outliers. Unless the dataset is sufficiently large, data split validation may result in significant variations in results across different splits of the data. In many cases, data splitting may not be sufficient to provide a good estimate of the generalizable performance of ML models.

#### Cross validation

Cross-validation is another widely employed approach to assess the performance of supervised models, especially when the available samples are limited in size [46]. Among the techniques of cross-validation and its variations, K-fold cross-validation is most frequently used [46]. In this method, the dataset is randomly divided into k equal-sized parts, where k is an arbitrarily chosen number. The model undergoes training k times, each time using k − 1 folds as the training set and the remaining fold as the validation set. This approach maximizes the amount of data used for training while ensuring that all data serves as part of the test set at least once, providing a more accurate estimate of performance. Leave-one-out is one variation, where the model is evaluated by leaving out just one data point from the dataset for testing, while the rest of the data points are used for training.

Typically, the results from each run of cross-validation are averaged to obtain a comprehensive cross-validation score [46]. However, examining the scores from each fold individually can be informative; significant discrepancies between them may signal issues such as outliers or class imbalances [47]. When results from different folds show considerable variability, it can be challenging to interpret the overall model performance. This high variability can result from the random sampling of data points into each fold. Nonetheless, cross-validation is a more robust method of model validation and should be preferred over a simple data split whenever possible, despite being computationally more demanding than data split validation.

#### External validation

External validation represents the most robust approach for gaining a genuine understanding of ML generalizability [45, 48]. This involves employing a different data set than the one used for model training and testing. This concept is exemplified in studies that employ ML models to identify subgroups with varying activity behavior patterns based on a wide range of individual, demographic, psychological, behavioral, environmental, and physical factors for intervention allocation and design [49–51]. While these models have proven successful in identifying target subgroups, it remains uncertain whether the same subgroups can be identified in a different population with potentially distinct cultural and country-specific influences on active behaviors. Validation on an external population can determine whether such ML models can serve as screening tools beyond the population they were originally trained and validated on.

### Performance metrics

**Fig. 5:**
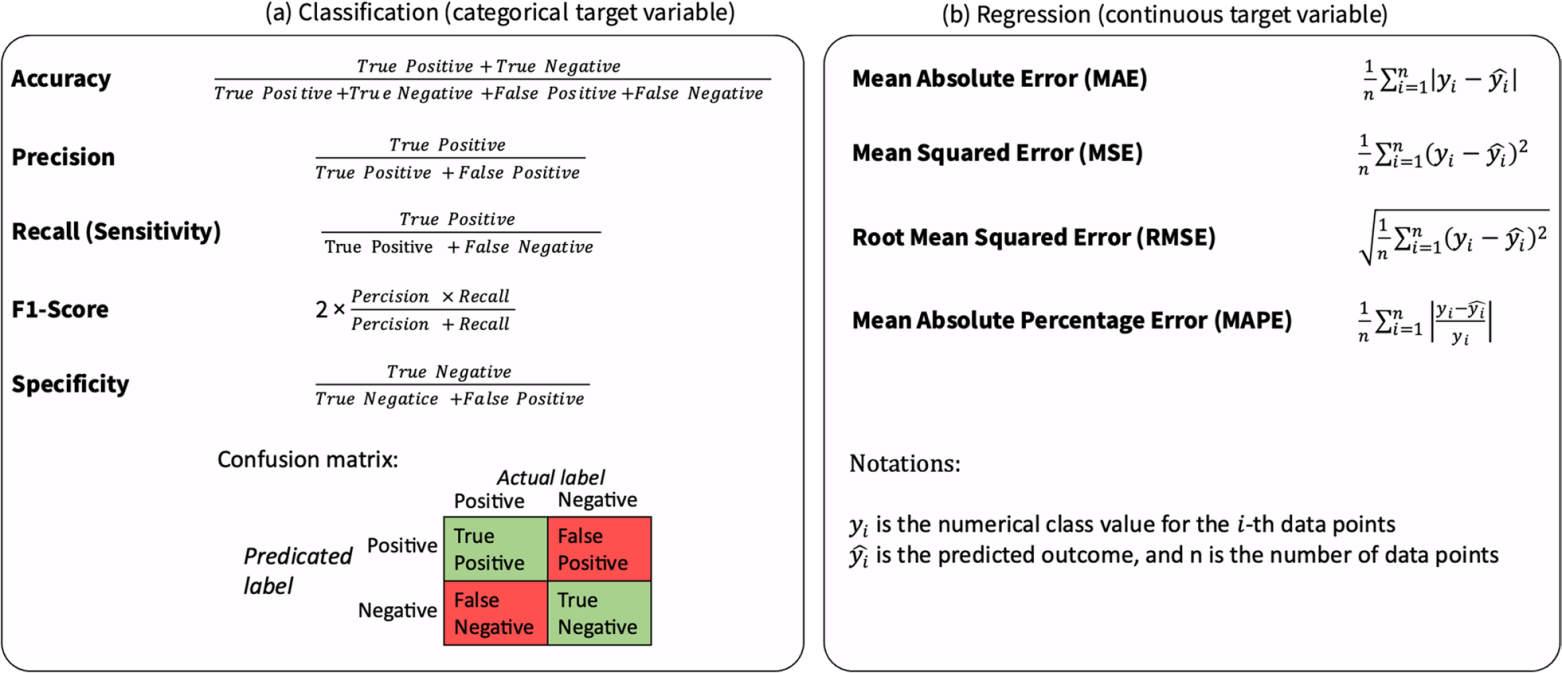
Performance metrics for evaluation of **a** classification and **b** regression problems

In supervised learning, the model’s performance can be objectively measured since the class variables are known for the data used for training, validation, and testing. Having these labels allows for the calculation of various quantitative metrics, offering an objective assessment of the model’s performance. An essential evaluation tool in supervised learning is the confusion matrix, which offers an overview of a supervised model’s performance by comparing its predictions to the actual values. A confusion matrix and commonly used performance metrics for classification tasks are illustrated in Fig. 5a.

The primary metrics used to evaluate classification models—accuracy, precision, and recall—can be derived directly from the confusion matrix [52]. Accuracy represents the most intuitive metric, showcasing the percentage of correctly classified observations overall. It offers a general overview of the model’s effectiveness but might not be sufficient in scenarios with imbalanced classes, where other metrics like precision and recall become vital for a deeper understanding of the model’s performance. Precision measures how often the ML model is accurate when predicting the target variable, while recall assesses the model’s ability to identify all instances of the target variable.

Commonly used performance metrics for evaluating regression models include the mean absolute error (MAE) and root mean squared error (RMSE). MAE quantifies the average magnitude of errors between predicted and actual values. It provides a straightforward measure of how far, on average, the predictions are from the actual values. On the other hand, RMSE offers an interpretable measure in the same units as the dependent variable. It considers the squared errors between predicted and actual values, which penalizes larger errors more than smaller ones due to the squaring operation. Lower RMSE values indicate better model performance. Other performance metrics that can be used for regression tasks are demonstrated in Fig. 5b.

Each metric reflects a different aspect of the model quality, and depending on the use case, ML practitioner should choose to examine the most appropriate one. Still, although quantitative assessment of performance is possible, the evaluation of supervised algorithms must be approached with caution [52]. For instance, solely reporting the total accuracy of a classification model—which measures the percentage of correctly classified data points—can be misleading in datasets with imbalances in the target variable’s classes. For example, consider a classifier tasked with predicting mortality based on variables describing the duration and timing of 24-h physical activity, sedentary, and sleep. Such a classifier might predict ‘negative’ for all data points and achieve high accuracy, not due to high predictive power but because a small proportion of the study sample experiences mortality. This demonstrates the importance of selecting appropriate performance metrics to properly assess the performance of the ML model.

## Unsupervised methods

Unsupervised learning is a type of ML where the algorithm makes use of unlabeled data. This means that unsupervised algorithms do not have specific target or output variables to learn from. Instead, these algorithms seek to find patterns, structure, or relationships in the data on their own, without any information guiding what is correct or incorrect [23, 53]. In general, the primary goal of unsupervised learning is to discover underlying patterns or hidden structures in the data. This can involve tasks such as clustering, where the algorithm groups similar data points together, or dimensionality reduction, which reduces the number of features in the data while preserving important information [14, 23, 53].

### K-means clustering

K-means clustering is an unsupervised ML method designed to partition the data into a user-defined number (K) of disjoint clusters based on the input variables (features) [54]. The aim is to maximize the similarity of data points within each cluster and minimize the similarity to data points in other clusters, according to the objective function. The K-means algorithm achieves this by initially selecting K centroids at random. Then, each data point is assigned to the nearest centroid, typically determined by Euclidean distance. The centroids of these clusters are recalculated, and the process of assignment and updating continues iteratively until the centroids stabilize or a user-specified maximum number of iterations is reached.

The objective function in the K-means algorithm is to minimize the within-cluster sum of squares (WCSS). The goal is to adjust the centroids to minimize the overall spread or variance within each cluster, resulting in the formation of more cohesive and distinguishable clusters. Mathematically, the WCSS is represented as below:

$${\text{WCSS}}={\sum }_{i=1}^{K}{\sum }_{x\in {C}_{i}}{\Vert x-{\mu }_{i}\Vert }^{2}$$

where $K$ is the number of the clusters, ${C}_{i}$ is i-th cluster, ${\mu }_{i}$ is the centroid or mean of cluster of ${C}_{i}$, and ${\Vert x-{\mu }_{i}\Vert }^{2}$ indicates the squared Euclidean distance between data point $x$ and centroid ${\mu }_{i}$

While K-means is favored for its simplicity and efficacy, its dependence on the initial random placement of centroids can potentially lead to inconsistent results across different executions [55]. Selecting the appropriate number of cluster groups is also a challenge when applying K-means. Techniques such as the elbow method and silhouette analysis are typically used to find the appropriate number of clusters and evaluate the inter-cluster separation distance [54].

### Principal component analysis (PCA)

Dimensionality reduction techniques are unsupervised methods that aggregate dimensions while attempting to preserve as much of the data’s structure as possible. This means that observations that are ‘close’ to each other in the original dataset remain so in the lower-dimensional representation. Principal Component Analysis (PCA) is a widely used linear dimensionality reduction method in which the new uncorrelated features are weighted linear combinations of the original data [56]. The objective of PCA is to identify directions (known as principal components) that maximize the variance of the data, capturing its most important features while reducing complexity. PCA works by decomposing the initial features into orthogonal components, each being a linear combination of the original features [56]. These components are evaluated based on the variance they capture from the original data, enabling the retention of only the most significant components and, consequently, reducing dimensionality.

The practical applications of PCA extend to various domains, such as visualization, noise reduction, and preprocessing data prior to its utilization in other ML algorithms [57]. However, due to the assumption of linearity in the data, PCA may not effectively capture the variance and could often yield suboptimal results. While the principal components are orthogonal, ensuring their statistical uncorrelation, this may hinder the interpretable meaning for each component when compared to the original features [56].

### Evaluations of unsupervised learning

Due to the inherent absence of explicit target variables in unsupervised methods, the objective evaluation of their outcomes becomes a challenging task [53]. Instead, evaluations of unsupervised methods are typically aimed at assessing the quality and meaningfulness of the discovered patterns or structures within the data. This requires evaluation measures, which assess the internal consistency and quality of the results.

**Fig. 6:**
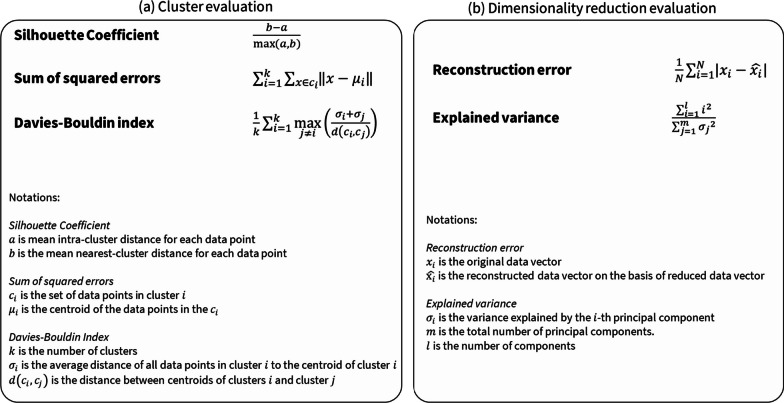
Performance metrics for evaluation of **a** clusters analysis and **b** dimensionality reduction

A number of evaluation metrics exist for clustering methods [53, 58]. The Silhouette Coefficient, for instance, quantifies the similarity of an object to its own cluster in comparison to other clusters, providing insights into the distinctiveness and coherence of clusters. Similarly, the Davies-Bouldin Index evaluates the average similarity ratio of each cluster with its most similar cluster, a metric for assessing the quality of cluster separation. When employing dimensionality reduction techniques such as PCA, two fundamental metrics, among others, are commonly considered [58]. These metrics include the reconstruction error, which measures the resemblance of the reduced-dimensional representation to the original data, and the explained variance, which quantifies the proportion of the total data variance retained in the reduced space [58]. Figure 6 illustrates the commonly used evaluation metrics for unsupervised learning.

## Challenges and considerations in machine learning modelling

### Overfitting and underfitting

ML modeling comes with inherent challenges, among which underfitting and overfitting are two prevalent issues. Underfitting occurs when the model inadequately captures the underlying patterns and distribution of the data [59]. As a result, the model fails to learn crucial characteristics, leading to poor generalization. Conversely, overfitting happens when a ML model learns the data too well, absorbing not just the fundamental patterns but also the noise or random fluctuations within the data [59]. Consequently, the model becomes excessively complex, finely tuned to the particular dataset it was trained on but lacking the ability to generalize, which diminishes its performance in real-world scenarios where variations in data distribution are typical.

**Fig. 7:**
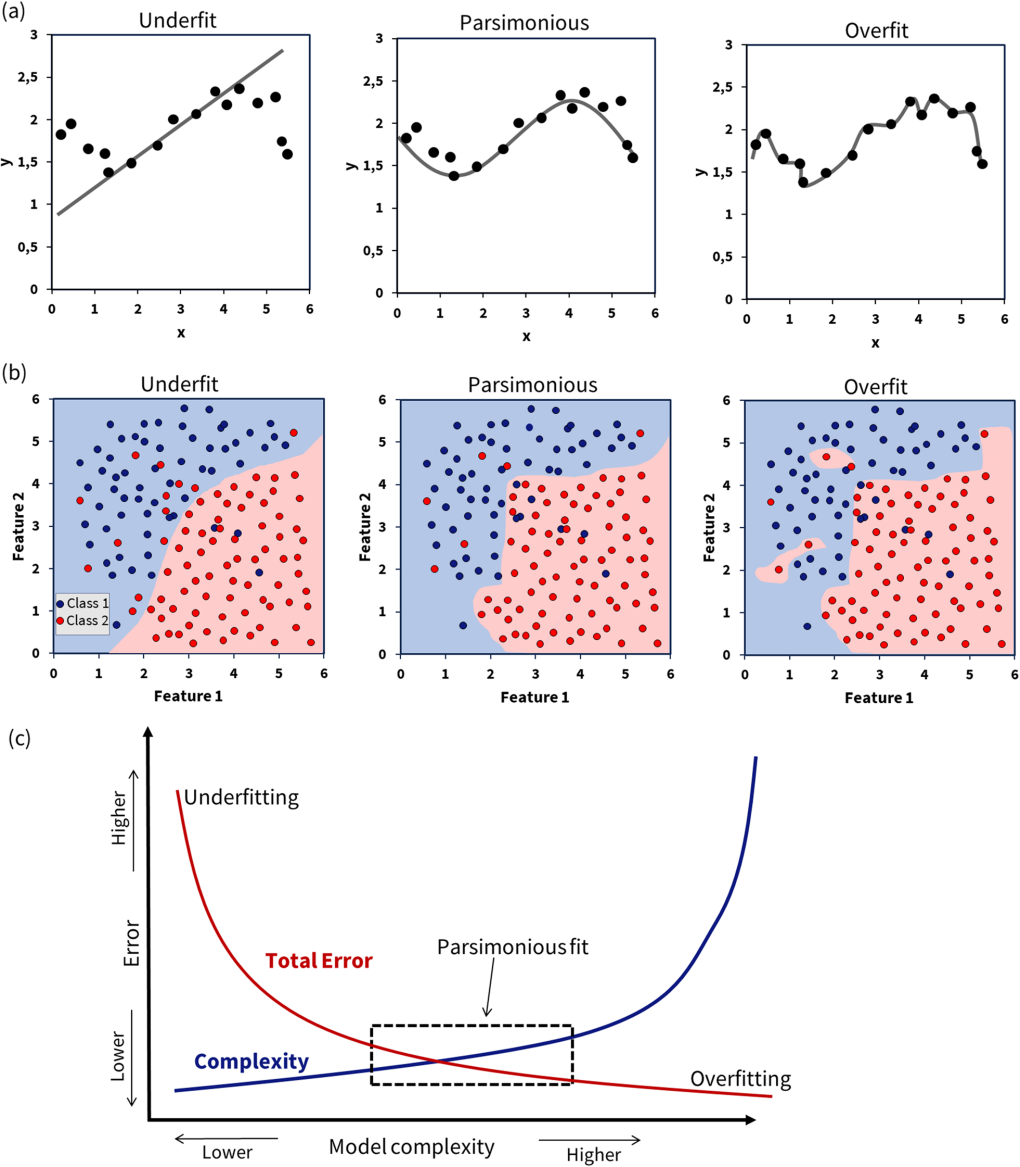
**a** A regression problem with randomly generated data points is depicted. Varying the hyperparameter changes the model’s complexity (flexibility), resulting in three distinct cases: underfitting (the model is too simple to describe the underlying process, exhibiting high bias and low variance), a parsimonious fit (representing the least complex model that effectively describes the observed data), and overfitting (where the model is overly complex and fits noise, leading to high variance and low bias). **b** A classification problem with randomly generated data points is depicted, showing a similar scenario to the regression problem. **c** This sketch illustrates the relationship between total error and complexity when training a ML algorithm. A model that is overly simplistic can lead to a high total error, primarily due to underfitting. Increasing the model’s complexity may help reduce the error rate, but it is essential to find a ‘sweet spot’. Finding the sweet spot depends on the problem at hand and how much error can be tolerated. Increasing the model’s complexity excessively leads to overfitting. The parsimonious fit is achieved when the error is minimized, and the model has a reasonable level of complexity

Consider, for instance, the development and validation of an ML model designed to estimate the number of steps a person takes based on data from wearable activity monitors. Overfitting might cause the model to register every minor movement as a step, leading to noisy predictions, while underfitting could result in a model that overlooks many actual steps. The ideal balance would be a model that performs well across various participants, especially those not included in the training data. Figure 7 demonstrates the problem of underfitting and overfitting in comparison to optimal model.

### The curse of dimensionality

Contrary to common belief, having an abundance of features does not necessarily enhance the performance and quality of ML tasks; in fact, it may adversely affect the performance and capability of ML algorithms [29, 60]. With an increase in the number of dimensions (or features), the data points tend to become sparser. Each data point is situated in a high-dimensional space where it is improbable for all possible combinations of features to be present in the data. This sparsity complicates the task for ML algorithms to discern and capture significant patterns and relationships within the data.

In a high-dimensional feature space—a dataset with hundreds or thousands of features—it is also likely that some features are interrelated or convey redundant information. Such correlated features and redundant information can pose problems for ML modeling, potentially degrading algorithm performance and, in some cases, leading to biased results and false pattern recognition. These issues, which escalate as the dimensionality increases, are collectively known as ‘the curse of dimensionality’ [60].

#### Addressing the problem of curse of dimensionality

The curse of dimensionality can be mitigated with both feature selection and dimensionality reduction [26, 27]. The choice could depend on a number of considerations such as types of data and the problem at hand. Imagine a study that aims to predict individuals’ engagement in regular exercise based on a multitude of factors. These factors could include demographic information (age, gender, etc.), lifestyle factors (diet, smoking habits), environmental factors (access to parks, gyms), psychosocial factors (motivation, self-efficacy), and even genetic markers, all of which could potentially be related to metabolism and physical activity. The goal of feature selection in this scenario would be to identify the most relevant and influential predictors of regular exercise from this extensive set of potential features. In the same scenario, dimensionality reduction could help to identify patterns and correlations among the multitude of features. It might reveal that certain lifestyle and environmental factors tend to cluster together, suggesting that a composite feature could be more relevant for modeling than the original features alone.

Another commonly used approach for mitigating the curse of dimensionality is feature selection guided by domain knowledge [25, 29]. For instance, consider a scenario where we are utilizing a ML approach to identify predictors of sitting time in office workers. While numerous predictors from various domains could potentially serve as inputs to the model, leveraging domain expertise can assist in prioritizing the selection of features that are relevant while considering feasibility and the potential for meaningful changes.

In this example, experts in occupational health and ergonomics might recommend prioritizing factors such as workstation setup, work schedule flexibility, and office ergonomics as primary considerations, while placing less emphasis on other relevant features that may be less relevant to workplace modifications, such as weight status, education, and diet. These experts understand that these aspects are not only linked to sitting time but also represent actionable variables that organizations can address to promote healthier work habits. By concentrating on such modifiable factors, the ML model becomes not only more precise in its predictions but also more actionable in terms of suggesting changes that can improve the well-being of office workers.

### Selecting the hyperparameters

ML algorithms rely on a set of parameters to operate effectively, known as hyperparameters. These parameters play a crucial role in determining the accuracy of the final models and can significantly impact the interpretation of the results [61]. The importance of selecting proper hyperparameters can be clarified through a clustering analysis example. For instance, consider a dataset of individuals’ sleep behaviors, where the goal is to identify distinct groups of individuals with varying sleep patterns—a task that can be performed with K-means clustering algorithm. Choosing the appropriate number of clusters for this problem is a critical decision, as it directly influences the granularity and interpretability of the results. Creating a higher number of clusters can lead to fine-grained distinctions among data points, potentially revealing subtle patterns within the data. However, having a high number of clusters makes it difficult to extract meaningful insights. Conversely, selecting too few clusters may oversimplify the analysis, obscuring important distinctions among groups. While the exact number of groups with distinct patterns of sleep behavior is not known a priori, the number of clusters is a hyperparameter that needs to be set before running the algorithm. Similarly, most ML algorithms also require the selection of several hyperparameters during model development and validation.

## Machine learning use cases in behavioral physical activity, sedentary, and sleep research

The current section provided a brief overview of three areas related to physical activity, sedentary, and sleep behaviors where ML has been widely employed. It is hoped that the readers will derive ideas and inspiration from the case studies, which they can then apply to their own research domains.

### Characterization and dimensions of movement and non-movement behaviors from wearable devices

In the era of wearable devices, ML has emerged as a powerful and robust tool for transforming motion signals gathered from these devices into various variables such as postures, activity type, intensity, and duration of physical activity, sedentary behaviors, and sleep, among others [7, 62–64]. Prior to the prevalence of ML techniques, analytical methods primarily focused on quantifying movement intensity and energy expenditure derived from wearable devices [8, 65]. Traditionally, the measurement and categorization of movement behaviors from wearable data relied heavily on conventional statistical approaches. However, with the integration of ML methodologies, there has been a significant improvement in the accurate measurement and classification of more complex movement behaviors and postures from wearables [8, 63]. Several studies have showcased the enhanced predictive abilities of ML in determining activity types, intensity levels, and energy expenditure compared to other statistical methods [7, 8, 63].

In addition to the improved prediction accuracy, ML-based models have been developed using relatively shorter time windows, typically ranging from 5 to 30 s, as opposed to the conventional 60-s intervals [8, 63]. The existing literature also suggests that ML holds the potential to address historical challenges linked to the different positioning of wearable devices on the body. For example, while wearable accelerometers worn on the thigh were once deemed essential for accurately identifying sit-to-stand transitions and sitting patterns, recent studies have revealed that ML can achieve similarly accurate classifications when applied to data from devices worn on the hip [66]. Consistent with this trend, a systematic review examining the predictive accuracy of ML models for activity prediction noted that ML algorithms may possess the ability to predict activity types accurately, irrespective of the location where the wearable accelerometer-based activity monitor is positioned on the body [8].

Both supervised and unsupervised algorithms have been widely utilized to translate wearable motion signals into variables representing physical activity, sedentary behaviors, and sleep behaviors [8, 53]. More sophisticated ML methodologies, such as ensemble methods that combine multiple ML algorithms, have been also proposed and validated for this purpose [33, 67]. Despite this success, there remains uncertainty about which ML algorithm is best suited for predicting movement behavior intensity, type, and energy expenditure. A notable drawback of existing studies is the development and validation of algorithms using limited data, typically involving few participants performing only a handful of activities [68]. Moreover, the accuracy of ML models is rarely assessed on an external dataset distinct from the one used for training [8]. Advancing the research in this field necessitates testing ML algorithms under fully free-living conditions, often termed as ‘in-the-wild’ scenarios. Future studies should prioritize assessing the generalization performance of ML algorithms on datasets that differ from those used for their training.

Newer research is leveraging the power of ML to automatically identify specific features that might exhibit correlations with physical activity, sedentary behavior, and sleep behaviors [69, 70]. This may eventually lead to more harmonized analysis of wearable data through automation and increased objectivity achieved by machine intelligence. Such innovative approach is exemplified in a recent study that employs an unsupervised methodology to independently uncover dimensions of accelerometry data that are closely linked with both sedentary behavior and physical activity [70]. While the precise relationship between these machine-learned variables and various health outcomes, as well as their practical applications, are areas that require further exploration, it is likely that such variables could potentially be a better predictor of health and diseases [69]. An early demonstration of the effectiveness of ML for learning directly from wearable data is highlighted in another recent study where numerous features extracted from accelerometer data were input into ML algorithms, leading to the remarkable prediction of Parkinson’s disease onset years before clinical diagnosis [71]. Future studies should consider whether ML applied to movement data measured by wearables can predict future health conditions and diseases.

### Profiling of movement and non-movement behaviors

A notable feature of ML, setting it apart from traditional statistics, is its capacity to generate novel hypotheses from data. Unlike traditional hypothesis testing where researchers formulate a specific hypothesis and then test it against data, ML can be used to derive and generate hypotheses from data by seeking patterns and trends [14]. This principle has already served as the foundation for applying ML, particularly through unsupervised clustering approaches, for the identification of previously unrecognized profiles of physical activity, sedentary, and sleep behaviors without having a predefined hypothesis [16, 19, 72, 73].

Studies employing ML approaches for data-driven profile discovery have so far identified a number of profiles characterized by distinct patterns, variations, and timing of movement and non-movement behaviors [16, 30, 74]. These machine-learned profiles appear to be important and linked to a number of health markers, even after accounting for total amount time spent in physical activity, sedentary behaviors, and sleep [16, 30, 72, 74]. A good example of such research is a recent study utilizing clustering analysis to construct multidimensional sleep profiles based on multiple accelerometer-measured sleep characteristics, reflecting sleep quantity, quality, schedule, variability, and regularity [75]. The study identified a total of five distinct sleep profiles, among which male individuals in the profile with sleep irregularity and variability exhibited elevated cardiometabolic risks.

Another significant aspect of ML approaches, which holds considerable potential for profile analysis, is the capability to merge diverse variables. This feature becomes particularly valuable when considering the integration of variables that may not all adhere to the same units of measurement, such as time. While in recent years there has been a tendency to utilize compositional data analysis approaches [2, 3, 76], such models remain to be primarily appropriate for examining the interdependent associations among physical activity, sedentary behavior and sleep—variables with same units of measurement. ML approaches can potentially be a better analytical tool for examining the potentially interdependent relationships among diet, lifestyle behaviors, physical activity, sedentary and sleep behaviors—a scenario where ML approaches can potentially be a better analytical tool. An illustrative case is seen in a recent study that employed 65 variables to characterize patterns of accumulation of sedentary time and sedentary breaks [72]. Through the utilization of the K-means clustering algorithm, the study identified four distinct profiles, each exhibiting unique patterns of accumulation of sedentary time and sedentary breaks. These differences were shown to be associated with markers of cardiometabolic health. Another example is a study that integrated 24-h physical activity, sedentary behaviors, and sleep with diet quality in the cluster analysis [77]. The identified profiles revealed that the combination of diet quality and use of time in a given day determines behavioral profiles that are significantly related to all-cause mortality.

One of the main challenges in applying ML to the data that are compositional in nature is that these approaches were not originally designed to handle compositional parts as a whole [78]. ML applied to compositional data still requires further development, and additional efforts are necessary to understand how to effectively utilize ML with such data. Thus far, there has been a tendency to rely on the K-means clustering algorithm and its variants for profiling physical activity, sedentary, and sleep behaviors [16, 19, 72, 79], likely due to their simplicity and efficiency. However, future research directions in profiling analysis should explore and assess alternative clustering algorithms. Current studies have generally been limited only to accommodate variables representing only physical activity, sedentary, and sleep behaviors without considering other lifestyle choices and health-related behaviors [16, 19, 70, 77]. This limitation presents an opportunity to explore the potential of ML in identifying clusters encompassing a broader spectrum of health-related behaviors that may better predict future health status and diseases.

### Machine learned correlates and determinants of activity, sedentary, and sleep behaviors

One of the most fundamental research questions related to human movement and non-movement behaviors is why some people choose to adopt healthier behaviors than others, such as being more active [80], experiencing better sleep [81], and spending less time in sedentary [82]. This question is particularly complex because these behaviors are influenced by a multilevel hierarchy of factors, as diverse as personality [83], financial incentives [84], weather conditions [85], among others [49]. Until now, the majority of previous studies studying the correlates and determinates of physical activity, sedentary, and sleep behaviors have employed classical statistical modeling, such as regression analyses [86, 87].

In classical statistics, these analyses tend to be limited by the data analyst’s decisions regarding how associations and interactions are hypothesized (knowledge-driven). This limitation arises because the factors chosen for inclusion in the analyses are primarily subjective, selected based on their conceptual relevance and, in some instances, initial empirical associations. Such analyses may hinder the identification of novel and innovative categories of correlates essential for advancing this field further [49, 88]. To overcome these limitations, embracing more flexible and exploratory methods that allow for the identification of possibly unforeseen correlations and interactions could be beneficial.

In particular, ML algorithms that generate hierarchical models appear to be ideally suited for addressing this research challenge. Multiple studies have employed decision trees [50], random forests [89], and other ML algorithms [90] to automatically seek through extensive lists of potential predictors and identify the most influential ones for physical activity, sedentary, and sleep behaviors. ML algorithms have also been used to construct an interconnected web of factors influencing these behaviors based on empirical data [49, 50, 90]. These studies have even revealed previously less established and unknown factors that hold the potential to explain these behaviors [49].

It seems that tree-based algorithms are most commonly utilized in research on correlates and determinants [49, 50], possibly owing to the transparency they offer in the final model. There exists a diverse spectrum of ML algorithms, in addition to hierarchical and tree-based models, that can potentially be applied in the research of correlates and determinants. This warrants further research into utilizing ML algorithms and approaches for correlate and determinant research, which may accordingly lead to the discovery of previously unrecognized associations and promoting the advancement of the field by uncovering novel insights.

For instance, ML can also be employed to address the causal questions—such as why some people choose to have healthier behaviors. An emerging field in ML known as causal ML offers a promising avenue for health research [91]. Such techniques can separate each causal pathway to measure the treatment effect of interest. Although still in its infancy, causal ML holds the potential to answer many challenging questions that have traditionally relied on theoretical approaches like ecological modeling [92]. As innovative ML methodologies continue to advance, it is likely that utilizing these techniques to analyze both movement and non-movement behavioral data will offer a more profound comprehension of human behavior.

## Concluding remarks

ML provides a hypothesis-free approach for modelling complex datasets when the interaction and interrelationships between measured variables are complicated. These methodologies overcome the constraints of many classical statistical models and offer an alternative option for making sense of physical activity, sedentary, and sleep behavior data, and generating innovative hypotheses from these data. We presented the ML modelling process, providing a detailed breakdown of each step to facilitate the application of ML techniques in the analysis of physical activity, sedentary, and sleep behaviors data. We also presented three areas of research where ML has been widely adopted and has achieved considerable success, demonstrating how ML may go beyond traditional statistics for addressing critical questions in physical activity, sedentary, and sleep behaviors research. ML will play a pivotal role in translating complex data sets into scientific knowledge and will become a proper addition to existing analytical tools.

Nowadays, thanks to advancements in high-level programming and the widespread availability of open-source languages such as Python and R, implementing ML through coding has become more accessible than ever [93]. ML algorithms are now commonly integrated into most statistical software and programs. Even those software platforms that are traditionally used for performing classical statistics now include some ML algorithms. ML tools can be broadly categorized into two groups: those that require at least some coding skills and those that offer a drag-and-drop interface. Table 1 shows a non-exhaustive list of software and statistical packages that can be used for ML modeling.

**Table 1:** List of software tools (non-exhaustive) and their characteristics available for machine learning modeling

Software tool	Open source	Drag-and-drop	Frameworks and libraries variety	Visualization variety
Python	Yes	No	High	High
R programming	Yes	No	High	High
Matlab	No	No	High	Medium
KNIME	Yes	Yes	Medium	Low
Weka	Yes	Yes	Low	Low
RapidMiner	No	Yes	Low	Low

Despite its popularity and success, ML has a few notable limitations that are important to consider. ML algorithms seek patterns in the data or learn the relationships that are optimal for predicting outcomes without a priori assumptions. While advantageous, this can introduce a conflict between the way humans and machines perceive these patterns [94]. Overall, ML models are typically considered as ‘black box’ approaches. In many applications, including research on physical activity, sedentary behavior, and sleep, understanding why a machine has learned to make its predictions may be more important than focusing solely on accuracy. Currently, explainable artificial intelligence (XAI) is an active field of research, focusing particularly on improving the transparency and interpretability of ML [94]. Nevertheless, the interpretability of ML approaches is still a concern in public and population health research.

Most recently, deep learning—a subfield of ML—has gained significant attention for its excellence in various supervised and unsupervised learning problems. Deep learning techniques focus on the use of neural networks with multiple layers—hence the term deep—to model and solve complex tasks [95]. A distinct characteristic that sets deep learning apart from other ML approaches is its ability to automatically learn and represent data features. Although the application of deep learning to the research of physical activity, sedentary behavior, and sleep is still emerging, these techniques have shown their versatility and effectiveness across various domains. For instance, deep learning has been employed in classifying movement behaviors [33] and designing physical activity recommender systems aimed at promoting active lifestyles [96]. We anticipate that as ML and deep learning techniques continue to evolve and demonstrate exceptional accuracy, their widespread adoption will significantly enhance their utility in analyzing and exploring device-estimated behaviors related to physical activity, sedentariness, and sleep.

## Data Availability

Not applicable.

## Abbreviations

ML: Machine learning
PCA: Principal component analysis
SVM: Support vector machines
